# The role of telemedicine for symptoms management in oral medicine: a retrospective observational study

**DOI:** 10.1186/s12903-022-02133-1

**Published:** 2022-03-27

**Authors:** Zahra Alsafwani, Caroline Shiboski, Alessandro Villa

**Affiliations:** Department of Orofacial Sciences, School of Dentistry, University of California San Francisco, 513 Parnassus Avenue, S- 722, San Francisco, CA 94143 USA

## Abstract

**Background:**

Severe acute respiratory syndrome coronavirus 2 has had devastating effect on access to care in many specialties and dental services including oral medicine. Following the shelter‐in‐place orders in March 2020, we implemented Tele(oral)medicine practices for the diagnosis and management of some oral medicine conditions.

**Objectives:**

To assess the role of telemedicine visits with respect to managing pain among patients affected by oral diseases.

**Methods:**

A retrospective chart review for all the new patients seen at their first visit via telemedicine between April 2020 and December 2020. The patient-reported pain score was recorded at each visit using a 0–10 scale. Differences in oral pain from the first fist to the follow-up visit of the patients were evaluated using the Wilcoxon signed-rank test.

**Results:**

A total of 137 new patients were included with a median age of 56 years. If seen in person, patients would have travelled a median distance of 65 miles. The most common oral conditions seen were reactive/inflammatory lesions. There was a 3-point median pain reduction from the first video visit to the first follow-up (*p* < 0.05) and a self-reported 65% median improvement of oral symptoms.

**Conclusion:**

Tele(oral)medicine was an effective method for symptoms management of oral medicine conditions.

## Introduction

Severe acute respiratory syndrome coronavirus 2 (SARS-CoV-2) has had devastating consequences globally, with multifaceted effects on education, and access to care in many specialties including dental and oral medicine services [1, 2]. Through the first months of the COVID-19 pandemic, a variety of medical treatments and visits were put on hold resulting in significant diagnostic delays and postponement of necessary medical and dental therapies [3].

On March 16, 2020, the American Dental Association (ADA) recommended that US dentists postpone all non-urgent dental procedures, and focus on managing dental emergencies only, in order to reduce patients’ potential exposure to COVID-19 infection [2]. According to an ADA Health Policy Institute survey conducted during the week of March 23, 2020, 76% of dental offices in the US were closed, although managing emergency patients, 19% were fully closed, and 5% were open but treating a lower number of patients [2].

In response to the pandemic most medical institutions in Unites State expanded the use of Telehealth practices as an alternative solution to continue patient care [4]. While Telemedicine was already largely utilized prior to the COVID-19 pandemic amongst several medical services (e.g., dermatology), the use in oral medicine and other dental practices was limited [4, 5]. Telehealth is defined as the use of communication technologies (e.g., computers and mobile devices) and digital information to access health care services remotely [6]. Telehealth services involve all healthcare professions (including healthcare professional education).

Teledentistry (a subset of telehealth, along with telemedicine) is the use of information technology to facilitate remote dental care, guidance, education, or treatment rather than direct face-to-face interaction with any patient [7]. A recent systematic review demonstrated that teledentistry is not a new concept and it has been known in the US military since 1994, but it was limited to consultations, diagnosis and treatment plan [8]. Teledentistry has been proven over the years to be beneficial for a remote dental screening, making diagnoses, providing consultation and proposing a provisional treatment plan until a face-to-face visit is possible [8].

Teledentistry has been used in several dental specialties. Sharma et al. reported that teledentistry can be beneficial in oral health education and promotion amongst children, and for the diagnosis, and monitoring of pediatric dental conditions [9]. Palmer et al. evaluated orthodontists' awareness of the use of digital and electronic technology, and found that over 70% of respondents agreed with the use of teletechnology and 36% expressed concern about security and privacy issues [10]. Moreover, Wood et al. investigated the demand for teledentistry among general dentists and oral and maxillofacial surgeons, concluding that teledentistry could be helpful in improving access to care and reducing healthcare costs [11].

According to the ADA teledentistry includes synchronous and asynchronous patient care, remote patient monitoring, and mobile health. The synchronous modality employs a virtual video visit to allow for a face to face encounter between the dentist and the patient, whereas the asynchronous approach focuses on the diagnosis and examination via data transfer of recorded health information (e.g., videos, radiographs, and intraoral photos) [12].

Following the shelter‐in‐place orders on March 16, 2020, we implemented Tele(oral)medicine practices for the diagnosis and management of some oral medicine conditions using synchronous and asynchronous modalities [13]. Tele(oral)medicine continued even after the shelter-in-place was lifted, especially for those patients who were not able to travel far, or those who wanted to avoid public transportation and maintain social distancing. The objective of the present study is to assess the role of telemedicine visits in managing pain recorded at the initial telemedicine visit and compared to the pain recorded at the first follow-up visit.

## Methods

### Study population and telehealth protocol

This study was a retrospective chart review of all new patients who were seen via telemedicine at the “Sol Silverman Oral Medicine Clinic” at the University of California San Francisco (UCSF) between April 1st, 2020, and December 22nd, 2020. The video visits were conducted by oral medicine specialists using Zoom (Zoom Video Communications, Inc.), a web conferencing platform that is routinely used at our institution for both instructional videoconferencing and for remote video visits. We used a standardized telehealth protocol developed by a team in our medical center, reviewed and approved by the Teledentistry Committee, and adapted for an Oral Medicine visit. As part of this protocol, which is described in detail in a prior publication [13], patients received detailed instructions on how to join the virtual visit via email prior to their appointment and were instructed to send intraoral photos (when available), any previous biopsies or previous health records related to their condition.

This study was approved by the UCSF Institutional Review Board (Protocol# 20-31367).

### Data collection

Clinical data were extracted from electronic medical records using a standardized data collection form and entered into an electronic spreadsheet. Specifically, we included demographic information, home Zip Code, referring doctor (and specialty), type of insurance, clinical diagnosis (based on the International Classification of Disease, 10th edition [ICD-10] codes), laboratory tests and imaging studies ordered at the time of the first video visit. Google Maps (www.google.com) was used to calculate the distance between the patient’s home and the oral medicine clinic. Patient-reported pain score was recorded at each visit using a 0–10 scale (0: no pain; and 10: the worst pain). At follow up video visits we also recorded the patient’s self-reported percentage of improvement since last visit.

### Statistical analysis

We used descriptive statistics (proportions for categorical variables, and were used to calculate median and range for continuous variables) to summarize of the patients’ agesocio-demographic characteristics (age, sex, race/thnicity, insurance status), presumptive clinical diagnoses, procedures ordered/performed, pain scale, percentage of self-reported percentage of improvement of oral symptoms and distance between the patient’s home and the oral medicine clinic. The presumptive diagnoses made upon the first visit were grouped into the following eight categories: (1) Reactive or inflammatory lesions, (2) Immune-mediated conditions, (3) Orofacial pain disorders (4) Infections, (5) Neoplasms, (6) Pre-neoplastic conditions, (7) Metabolic disorders and (8) Other.

Differences in patient-reported oral pain scores between first and follow up visits were evaluated using the Wilcoxon signed-rank test. The *p-*value was considered statistically significant if < 0.05.

## Results

### Patient characteristics

A total of 137 new patients were seen as part of telemedicine consultation for their first visit from April 1, 2020, to December 22, 2020 with a median age of 56 years (range 3–89 years; Table 1). The majority of patients were females (n = 79; 57%), and among 85 patients who chose to report their race/ethnicity, the majority (n = 70; 82%) reported being White.

**Table 1:** Demographics and insurance coverage among 137 new patients seen through a tele(oral)medicine visit from April 1 to December 22, 2020

Demographics and insurance coverag	N	(%)^a^
**Sex**		
Female	79	57
Male	58	43
Median age = 56 years (range 3–89 years)		
**Race/ethnicity**		
White	70	51
Asian	12	9
Hispanic	3	2
African American	1	1
Unreported	51	37
**Type of insurance**		
Private medical insurance	92	67
Medicare	31	23
Private dental insurance	4	3
DentiCal	5	4
Uninsured	5	4

^a^Percentages may not add to 100% due to rounding. Typically, percentages 0.5 or above are rounded up, and 0.4% or below are rounded down

### Type of insurance

For most patients (n = 92; 67%) their telehealth visit was covered by private medical insurance, followed by Medicare (n = 31; 23%), dental insurance (Private dental insurance, n = 4; 3%, Dentical, n = 5; 4%), and five patients (4%) were uninsured.

### Oral medicine referral

More than half of the patients (n = 82; 60%) were referred by medical doctors, with the greatest proportion coming from primary care physicians (n = 47; 34%; Table 2), followed by otolaryngologists (n = 17; 12%), oral maxillofacial surgeons (n = 5; 4%), dermatologists (n = 5; 4%), pediatricians (n = 3; 2%), immunologists and oncologists (n = 2; 2%). Twenty-one patients (15%) were referred by their general dentist and 35 (26%) were self-referred. If seen in person, patients would have traveled a median distance of 65 miles (range: 0.9–100 miles).

**Table 2:** Referring doctors for 137 new patients seen through a tele(oral)medicine visit from April 1 to December 22, 2020

Referring doctors	N	(%)
Dentist	21	15
Medical doctors		
Primary care physician	47	34
Otolaryngologist	17	12
Oral maxillofacial surgeon	5	4
Dermatologist	5	4
Pediatrician	3	2
Immunologist	2	1
Oncologist	2	1
Self-referred	35	26

### Diagnostic tests ordered

One third of patients (n = 51; 37%) required an oral biopsy (incisional biopsy: n = 32); excisional biopsy: n = 19) and were asked to schedule an appointment for an in-person visit. Panoramic radiographs and laboratory studies were ordered in 13 (9%) and three (2%) patients, respectively (Table 3).

**Table 3:** Diagnosis category among 137 new patients at their first tele(oral)medicine visit from April 1 to December 22, 2020

Diagnosis category	N	(%)	Presumptive diagnosis n (%)
Reactive	56	40	Fibroma, papilloma, pyogenic granuloma: n = 37 (52%) Hypersensitivity reactions: n = 2 (3%) Other: n = 17 (30%)
Autoimmune	31	23	Lichen planus: n = 14 (45%) Pemphigus/MMP: n = 3 (10%) RAS: n = 14 (45%)
Orofacial Pain	18	13	Burning mouth syndrome: n = 16 (89%) TMJ: n = 1 (5.5%) Myofascial pain: n = 1 (5.5%)
Infection	17	12	Oral candidiasis: n = 8 (47%) Bacterial infection: n = 1 (6%) Recurrent HSV infection: n = 2 (12%) Other: n = 6 (35%)
Neoplasm	9	6	SCC: n = 3 (33%) Dysplasia: n = 6 (67%)
Other	4	3	Pre-radiation
Metabolic	1	1	IBD related oral ulcer
Pre-neoplastic	1	1	Proliferative leukoplakia

SCC, squamous cell carcinoma; IBD, inflammatory bowel disease; MMP, mucous membrane pemphigoid; RAS, recurrent aphthous stomatitis

### Diagnosis and symptoms

The most common presumptive diagnoses made were reactive/inflammatory lesions (40%; Table 4), followed by immune-mediated conditions (23%), orofacial pain disorders (13%), infections (12%), neoplasms (6%), other (3%), and metabolic and pre-neoplastic conditions (1%).

**Table 4:** Diagnostic and laboratory tests ordered among 137 new patients at the time of at their first tele(oral)medicine visit from April 1 to December 22, 2020

Diagnostic and laboratory tests	N	(%)
**Biopsy needed**		
Yes	51	37
Incisional	32	63
Excisional	19	37
No	86	63
**Imaging studies needed**		
Panoramic radiograph	13	9
No	124	90
**Laboratory investigations needed**		
Yes^a^	3	2
No	134	98

^a^CBC was ordered for two patients; PT, PTT and INR were ordered for one patient

PT, Prothrombin time; PPT, partial thromboplastin time; INR, International normalized ration; CBC: complete blood count

### Symptoms management

When pain was considered, there was a 3-point median pain reduction (on a 1–10 scale) from the first video visit to the first follow up (5.5. vs. 2.5; p < 0.05), and a self-reported 65% (IQR = 50%-90%), median improvement of oral symptoms. Of note, sixteen patients (12%) did not report any pain at first visit and were therefore excluded from this analysis.

## Discussion

Due to the current ongoing SARS-CoV-2 pandemic, teledentistry usage has increased as a way to provide safe access and delivery of dental care. In response to the pandemic, several North American Oral Medicine practices established tele(oral)medicine services to provide virtual patient care and clinical education continuity for dental and oral medicine trainees [14]. Tele(oral)medicine has been proven to be an effective tool to assess oral mucosal disorders in a timely manner and address orofacial pain conditions, or postoperative complications that may not necessarily require an in person consultation thus reducing the risk of potential exposure to COVID‐19 [15]. Similarly, our study confirmed that tele(oral)medicine during the COVID-19 pandemic was a successful tool to manage the symptoms of a variety of oral mucosal diseases. We found that there was a significant reduction in oral pain between the first video consultation and follow-up with a 65% self-reported improvement of oral symptoms.

Teledentistry has been also used in other dental specialties. A recent study from Sharma et al. showed that teledentistry was a helpful tool to manage pediatric patients with limited access to pediatric dentists, monitor dental conditions, conduct screening programs, and promote oral health in children [9]. Rollert et al. aimed to evaluate the effectiveness of telemedicine consultation for preoperative assessment of oral and maxillofacial surgery patients and showed that all patients were assessed correctly during the virtual consultation [16].

Most patients with oral mucosal conditions see several providers before having a correct diagnosis and travel long distances due to a paucity of oral medicine specialists in the United States [17]. The majority of oral medicine specialists work in academic settings in urban areas making it challenging for patients living in rural areas to easily access oral medicine services (e.g., dental schools or academic medical centers) [17]. A study from Brigham and Women’s Hospital in Boston, MA showed that patients traveled a median distance of 18.9 miles (range 0.2–525) to see an oral medicine specialist, with over 85% living within 60 miles away from the oral medicine clinic [17]. Similarly, another study conducted at the University of Alberta, Canada showed that the average distance traveled by patients to access the oral medicine clinic was 55.5 km (34.5 miles) and the average wait time for the patients to be seen was 105.5 days [18]. In our study, patients would have traveled a median distance of 65 miles (range: 0.9–100) if they had been seen in person. Since traveling long distances may result in increased costs to patients, Telemedicine offers a unique opportunity for patients who otherwise do not have an oral medicine specialist in the vicinity.

In our study, more than half of the patients were referred by physicians (60%). Similarly, Villa et al. (2015) in another US study showed that two-thirds of the patients were referred by physicians and the remaining one-third referred by dentists (22%) [17]. This was different from the study by Friesen and colleagues which showed that 81% of the oral medicine patients were referred by dental practitioners with the general dentist being the most common (74.5%) [18]. Similarly, another study characterizing an oral medicine practice at a dental hospital in the United Kingdom showed that nearly three quarters (75%) of the patients were referred by dentists [19].

In our study, when the patient’s insurance was considered, two thirds of our patients (67%) had private medical insurance, and (23%) had Medicare. Similar results were reported by Villa et al. and showed that the most patients (66%) had private medical insurance, with (16%) having Medicare coverage and (5.7%) having Medicaid; (11%) of patients had a mix of public and private coverages, with the remaining (0.8%) being uninsured [17]. Tele(oral)medicine charges remain similar to in person visits although patients do not have to pay for transportation.

The most common oral conditions specifically seen were reactive lesions (40%) followed by immune-mediated conditions (23%), and orofacial pain disorders (13%). A biopsy was ordered for 37% of the patients. This was similar to what has been reported in the past for in-person oral medicine visits in other practices in the US. Specifically, Villa et al. showed that the most common diagnoses included immune-mediated mucosal conditions (27%), orofacial pain disorders (25%), benign tumors or neoplasms (10%), and dysplasia and cancerous conditions (7.6%), oral biopsy was the most common procedure performed [17]. In addition, Friesen et al. reported that the most common conditions seen were red and white lesions (38%) and immune-mediated disorders (29%) [18].

During the recent COVID-19 pandemic teledentistry has been used as a remote facilitator of dental treatment, guidance and education and offered a novel solution to continue dental practices during the pandemic [7]. On the other hand, there has been several challenges around acceptance of teledentistry by the dental providers and patients who need urgent care due to the lack of the actual dental procedures [7]. Especially at this time, reliance on telemedicine has grown, and recent studies have shown that patients are usually satisfied with telehealth [14]. A study conducted in New York showed an 8729% increase in video visit use during the COVID-19 period compared to the pre–COVID-19 pandemic [20]. Our previous work showed that oral medicine patients were pleased with Tele(oral)medicine sessions (85%) [14]. Moreover, a study from Ghai at el. reported that acceptance of teledentistry has been increasing day by day by patients and health care providers [7]. Patient satisfaction with video visits is high and does not seem to be a barrier toward a paradigm shift away from traditional in-person clinic visits [20].

Our study has some limitations. This was a single study center study within a large academic medical center, as such it may not be generalizable to other oral medicine practices in the US. Future studies should include other private and academic centers. Furthermore, we were not able to look at the reimbursement of telemedicine visits and compare it to in-person consultations. The Center of Medicare Services (CMS) in the US reported that due to the COVID public health emergency, telemedicine visits would be reimbursed at the same level as in-person services. However, this may change in the future.

## Conclusion

Tele(oral)medicine plays a valuable role in symptoms management of oral medicine related conditions, with several advantages over in person visits during the COVID-19 pandemic. It helps to facilitate public health mitigation strategies during the pandemic by increasing social distancing and saves patient time and costs associated to transportation use. Furthermore, remote access to oral medicine services may increase participation for those who are medically or socially vulnerable or who do not have ready access to providers such as patients with a special need or an elderly patient who need transportation. Tele(oral)medicine may be continued to be used in the future for an initial screening for oral mucosal conditions and to improve access to care to those patients that do not have an oral medicine specialist in their area.

## Declarations

### Availability of data and materials

The datasets analyzed during the current study are not publicly available due to include the patient’s information’s but are available from the corresponding author on reasonable request.
